# Type 2 diabetes subtypes for precision medicine: methodological challenges and alternative prediction-based approaches

**DOI:** 10.1007/s00125-026-06802-6

**Published:** 2026-07-25

**Authors:** Tim Mori, Christian Herder, Pedro Cardoso, John M. Dennis, Oliver Kuß

**Affiliations:** 1 Institute for Biometrics and Epidemiology, German Diabetes Center, Leibniz Center for Diabetes Research at Heinrich Heine University Düsseldorf, Düsseldorf, Germany; 2 German Center for Diabetes Research (DZD), Partner Düsseldorf, München-Neuherberg, Germany; 3 Institute for Clinical Diabetology, German Diabetes Center, Leibniz Center for Diabetes Research at Heinrich Heine University, Düsseldorf, Germany; 4 Department of Endocrinology and Diabetology, Medical Faculty and University Hospital Düsseldorf, Heinrich Heine University Düsseldorf, Düsseldorf, Germany; 5 Institute of Clinical & Biomedical Sciences, University of Exeter Medical School, Exeter, UK; 6 Centre for Health and Society, Faculty of Medicine, Heinrich Heine University Düsseldorf, Düsseldorf, Germany

**Keywords:** Classification uncertainty, Clusters, Limitations, Methodology, Precision medicine, Prediction models, Review, Subtypes, Type 2 diabetes mellitus

## Abstract

Growing interest in precision diagnostics has stimulated efforts to classify type 2 diabetes into more granular subtypes. Recent research has focused on four pathophysiology-based subtypes: severe insulin-deficient diabetes (SIDD); severe insulin-resistant diabetes (SIRD); moderate obesity-related diabetes (MOD); and moderate age-related diabetes (MARD). While these subtypes provide an attractive framework for precision medicine, they also come with methodological challenges. This narrative review highlights three key issues: (1) uncertainty about the existence of four discrete subtypes; (2) low certainty in subtype assignment; and (3) limited evidence for prognostic and therapeutic value compared with other approaches. As an alternative to subtyping, we discuss individualised prediction models, which provide personalised treatment recommendations based on routine clinical features. These models avoid some of the limitations of discrete subtyping but they do not provide insights into a person’s underlying pathophysiology. Thus, these two approaches can be viewed as complementary, each serving a different purpose in precision medicine.

**Graphical Abstract:**

**Figure:**
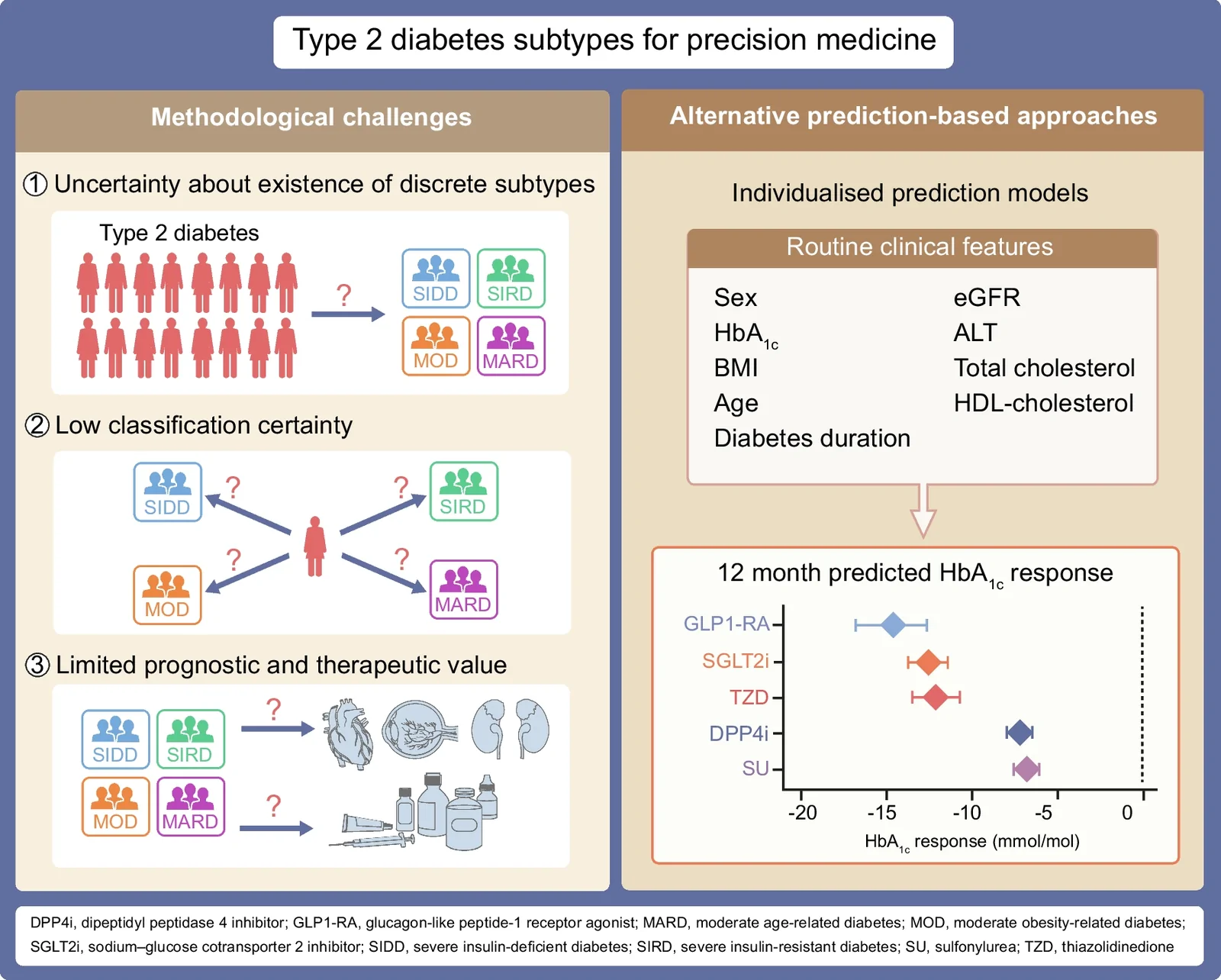

**Supplementary Information:**

The online version contains a slideset of the figures for download available at https://doi.org/10.1007/s00125-026-06802-6.

## Precision medicine and type 2 diabetes subtypes

Type 2 diabetes is a heterogeneous disease and its clinical presentation differs considerably between individuals [1–3]. For example, it is well established that some individuals with type 2 diabetes are living with obesity and are insulin resistant, whereas others are rather lean and insulin deficient [4]. Despite such differences, current guidelines for the management of type 2 diabetes typically employ a one-size-fits-all approach with few personalised recommendations [5]. Given the growing interest in precision medicine (see Text box, ‘Precision medicine’), recent research has focused on classifying diabetes into subtypes (or clusters, phenotypes or endotypes) that are more granular than the current type 1 and type 2 distinction [1, 6–9].

**Figure:**
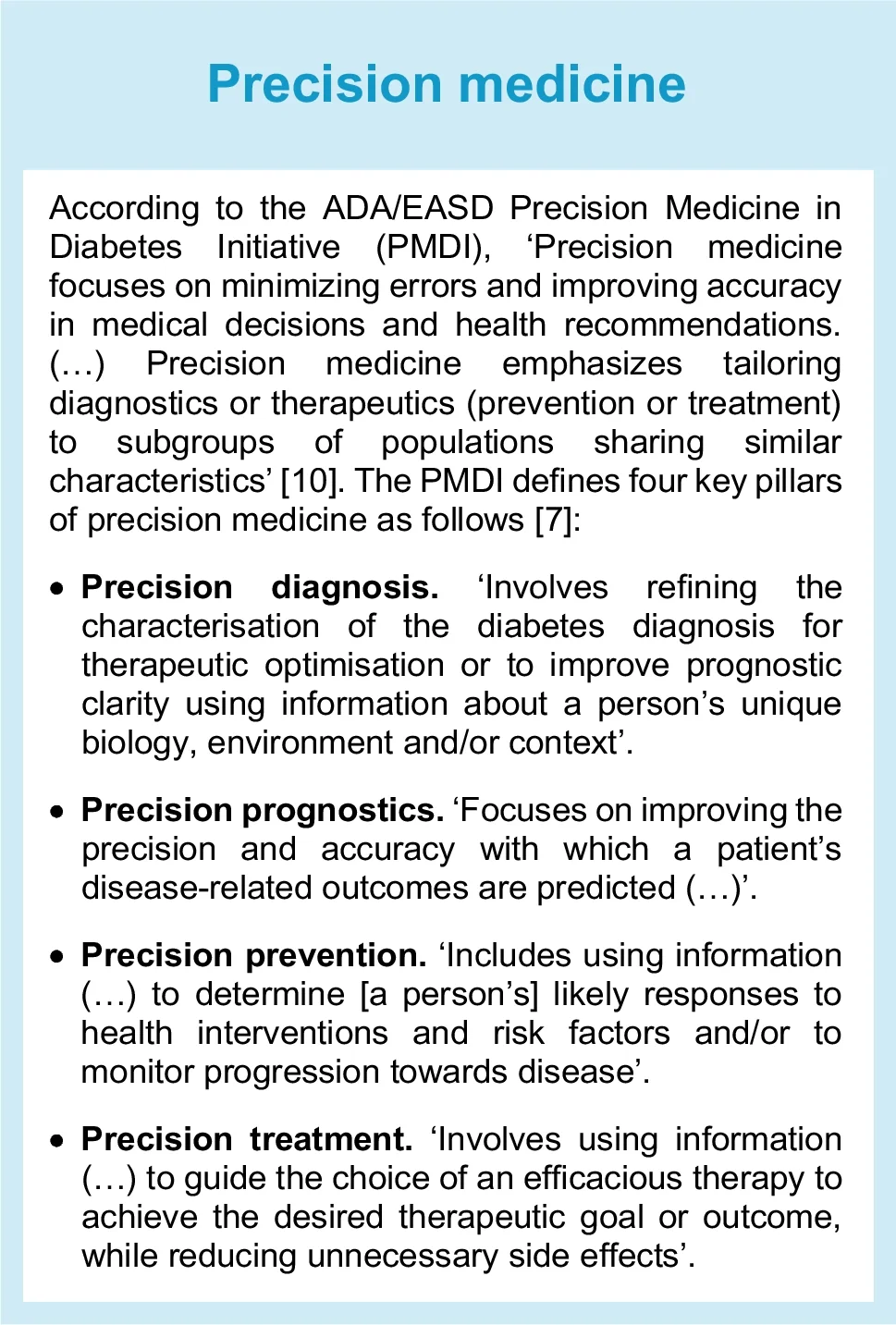

In their seminal paper published in 2018, Ahlqvist et al [8] used an exploratory clustering approach to show that newly diagnosed adult-onset diabetes (including both type 1 and type 2 diabetes) could be classified into five different subtypes (or subgroups). Their precision diagnosis approach was based on data-driven hard clustering using six clinical features: GADA, HbA_1c_, BMI, age at onset of diabetes, HOMA2-B and HOMA2-IR. HOMA2-B and HOMA2-IR are proxy measures of insulin secretion and insulin resistance, which are computed from fasting glucose and fasting insulin or C-peptide concentrations [11]. The five subtypes were labelled and described as follows [8, 12]:

1. severe autoimmune diabetes (SAID): GADA positive; early disease onset; low BMI; insulin deficient; high HbA_1c_
2. severe insulin-deficient diabetes (SIDD): GADA negative; otherwise similar to SAID
3. severe insulin-resistant diabetes (SIRD): GADA negative; late disease onset; high BMI; high insulin resistance
4. moderate obesity-related diabetes (MOD): GADA negative; early disease onset; high BMI; low insulin resistance (also known as ‘mild’ obesity-related diabetes)
5. moderate age-related diabetes (MARD): GADA negative; late disease onset; low BMI; low insulin resistance (also known as ‘mild’ age-related diabetes)

**Fig. 1:**
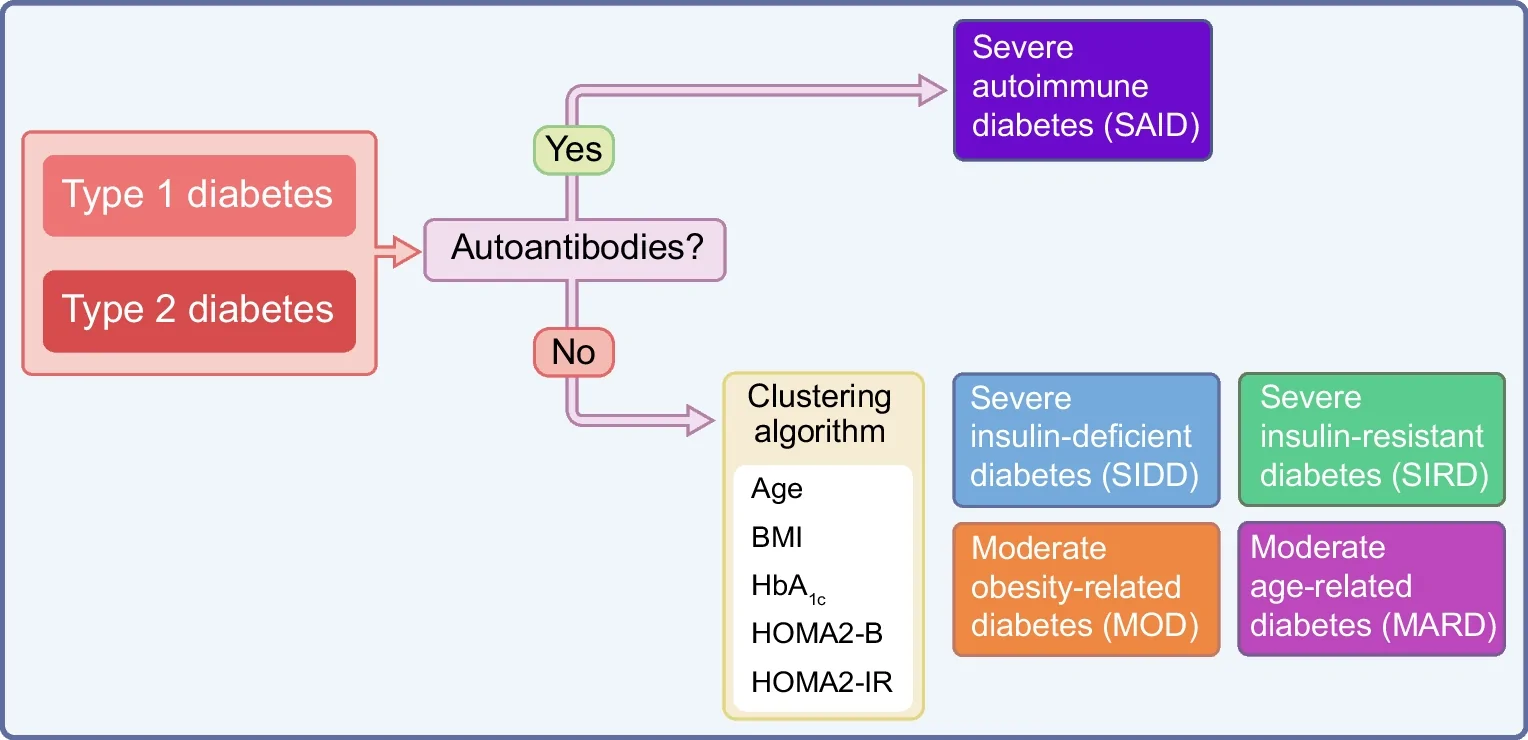
Overview of the precision diagnostic approach of Ahlqvist et al [8]. SAID corresponds to adult-onset type 1 diabetes (due to the presence of autoantibodies), whereas SIDD, SIRD, MOD and MARD correspond to subtypes of type 2 diabetes derived using a data-driven clustering algorithm. Note that the clustering algorithm (*k*-means) was applied only to individuals with type 2 diabetes (i.e. those without autoantibodies). This figure is available as part of a downloadable slideset

In this framework, adult-onset type 1 diabetes (typically GADA positive [1]) corresponds to SAID, whereas type 2 diabetes is subdivided into SIDD, SIRD, MOD and MARD. Thus, the key innovation lies in the more granular subclassification of type 2 diabetes (see Fig. 1) [6, 7, 13]. Regarding precision prognostics, several studies have since shown that the subtypes differ in their risk of glycaemic, microvascular and macrovascular outcomes [6, 8, 9, 14]. For example, individuals with SIRD may have the highest risk of chronic kidney disease, whereas those with SIDD may have the highest risk of retinopathy.

The proposed diabetes subtypes provide an attractive framework for type 2 diabetes precision medicine. However, discrete subtyping (i.e. hard clustering) also comes with methodological limitations that are sometimes overlooked. The aim of this narrative review is to raise awareness of these limitations by highlighting three key issues: (1) uncertainty about the existence of four discrete aetiological subtypes; (2) low certainty in subtype assignment; and (3) the limited evidence for prognostic and therapeutic value compared with other approaches. As an alternative to subtyping, we discuss recently proposed individualised prediction models, which aim to inform on precision treatment by providing personalised treatment recommendations based on individual patient-level clinical features.

## Methodological challenges of type 2 diabetes subtyping

### Uncertainty regarding the existence of four discrete subtypes

A frequently cited argument in favour of the four type 2 diabetes subtypes is that they have been replicated in several independent studies across diverse populations [6, 9]. Initially, Ahlqvist et al [8] replicated the diabetes subtypes in three Scandinavian cohorts. According to the Precision Medicine in Diabetes Initiative (PMDI) systematic review on subclassification of type 2 diabetes [6], the subtypes have since been directly replicated in at least 22 studies that included around 88,000 individuals from diverse ethnic and ancestral origins (e.g. in German [14], North American [15], Chinese [15] and Japanese [16] cohorts).

However, there is substantial methodological variability in how these different studies aimed to replicate the four type 2 diabetes subtypes. Varghese et al [17] distinguish between three different levels of replication. The first level directly uses the cluster coordinates identified in the original Swedish cohort (All New Diabetics in Scania [ANDIS]) and applies them to another population (e.g. [14, 18]). This has also been referred to as the ‘nearest centroid approach’ [14]. The second level involves de novo clustering in a different population but with the a priori assumption that there are exactly four discrete subtypes (e.g. [15, 19]). In *k*-means cluster analysis, this corresponds to having a pre-defined *k*-value that determines the number of clusters (i.e. *k*=4). The third level involves de novo clustering without such an a priori assumption, therefore allowing a different number of clusters to emerge from the analysis (e.g. [16, 20]).

The first two approaches automatically presume the existence of four discrete subtypes and therefore do not replicate the cluster analysis as a whole. The most convincing evidence would stem from the third approach, with confirmation in independent studies that four clusters provide an ideal subclassification of type 2 diabetes. However, in their literature review, Varghese et al [17] found that only 18 of 43 replication studies (42%) followed the third approach. Of these 18 studies, only seven (39%) were able to successfully replicate the four type 2 diabetes subtypes and the remaining 11 studies either identified different subtypes or failed to identify all of the subtypes proposed by Ahlqvist et al [8]. For example, Christensen et al [20] found only three instead of four type 2 diabetes subtypes when applying the same clustering procedure (i.e. *k*-means clustering with the same variables) in a Danish cohort.

However, it should be noted that not all of these replication studies had access to all five continuous clustering variables used by Ahlqvist et al [8]. In particular, HOMA2 measures were unavailable in four out of 18 studies (22%) [17]. Indeed, there has been considerable interest in replicating the diabetes subtypes with more readily available clinical features (i.e. without HOMA2 measures) [6, 21–24]. For example, Lugner et al [21] performed *k*-means clustering on data from the National Diabetes Register of Sweden using a different set of variables. Instead of HOMA2 measures they included six other routinely measured clinical features (systolic BP, diastolic BP, HDL-cholesterol, LDL-cholesterol, triacylglycerols and eGFR). However, a recent study suggests that such modified sets of clustering variables may not be suitable for identifying the four subtypes using de novo clustering [23]. In particular, HOMA2 measures appear to be indispensable for identifying individuals with SIRD [23, 25]. Therefore, replication studies with modified sets of clustering variables cannot provide definitive evidence for or against the four diabetes subtypes proposed by Ahlqvist et al [8]. Nevertheless, they illustrate that the results of diabetes cluster analyses are not unequivocal but are dependent on the specific input variables, clustering algorithm and population [6].

From a methodological perspective, a challenge of the subtypes is that they were derived from a data-driven cluster analysis (analogous to unsupervised learning), meaning that no ‘ground truth’ was available [26]. In other words, there is no way of knowing whether the proposed diabetes clusters correspond to true biological subgroups of diabetes, or whether they arise because a set of interdependent variables (e.g. BMI, insulin resistance, HbA_1c_) is entered into the clustering algorithm. In this sense, clustering can be seen as another way of capturing correlations between clinical features. However, unlike correlation analysis, it does not provide inferential tools like CIs or *p* values [27, 28]. Instead, cluster analysis relies on heuristic methods such as elbow plots or gap statistics to determine the optimal number of clusters from the data [27]. In other words, while cluster analysis is a useful tool for grouping similar data points together, its utility for discovering biologically distinct diabetes subtypes remains unclear.

To assess whether the proposed subtypes could be driven by correlations between clinical features, rather than by an underlying subgroup structure, Aoki et al [29] carried out a simulation study. They simulated a virtual patient dataset without any subgroups but preserving the correlation structure between the clustering variables. The assumed correlations were based on empirical data from the multinational SAVOR-TIMI 53 cardiovascular outcome trial [30]. For example, they included a strong positive correlation (*r*=0.5) between HbA_1c_ and fasting glucose (used for HOMA2 calculation) and a moderate negative correlation (*r*=−0.25) between HbA_1c_ and age at diabetes onset [29]. When they applied *k*-means clustering to these simulated data, the algorithm still reproduced the four subtypes (SIDD, SIRD, MOD and MARD), even though no discrete subgroups were present. A limitation of these findings is that the empirical basis for the virtual patient dataset (the SAVOR-TIMI 53 trial) might not be entirely suitable for studying the diabetes subtypes. In particular, the simulation was based on a multi-ancestry population with long-standing diabetes (mean of 8.7 years) and established CVD. Moreover, as the findings are based on a simulation study, they cannot be interpreted as definitive evidence against the existence of discrete subtypes. However, they illustrate that data-driven diabetes clusters could, in theory, be driven by correlations between clinical features rather than by discrete biological subgroups.

Taken together, the findings presented above suggest that there is considerable uncertainty regarding the existence of four discrete subtypes of type 2 diabetes. Nevertheless, the subtypes still appear to capture meaningful and clinically relevant differences in diabetes pathophysiology [8, 14, 31]. Aetiological differences between subtypes are considered further in the discussion section.

### Low certainty in subtype assignment

As well as the general uncertainty regarding the existence of four discrete subtypes, there is also uncertainty at the individual level when assigning people to these subtypes. While each individual with type 2 diabetes can technically be assigned to a subtype using the clustering algorithm, not every individual will match with that subtype equally well [2, 32]. Even in the original publication of Ahlqvist et al [8], a close examination of the distribution of clinical features reveals large variability within each subtype. For example, not every individual with SIRD shows severe insulin resistance and not every individual with SIDD exhibits reduced insulin secretion. In line with this, Pearson [2] argues that the phenotypic variability of type 2 diabetes may be difficult to capture with just four categories and that individual classification probabilities may be low.

In practice, this means that an individual’s clinical profile may lie between two subtypes (e.g. sharing similarities with both MOD and MARD) [25, 33] or not match any of the four subtypes [2]. For an illustrative example of borderline cases falling between two subtypes, consider the two individuals shown in Table 1. The clinical profiles of the two individuals are almost identical, except for a 0.5 kg/m^2^ difference in BMI. Yet, the clustering algorithm (nearest centroid approach based on the cluster coordinates from the ANDIS cohort [8]) assigns individual A to the MARD subtype and individual B to the MOD subtype. For such individuals, the subtype assignment is unstable, as small changes in clinical variables may lead to a different subtype. Consequently, their classification certainty is low and subtype-specific risk predictions may be less reliable. Moreover, measurement error as well as longitudinal biological changes (e.g. in HbA_1c_ or BMI) may lead to changes in subtype assignment [34]. For example, in the German Diabetes Study almost one out of four individuals changed their subtype within 5 years after diagnosis [14]. Besides natural disease progression, subtype assignment may also change in response to treatment, as some of the cluster variables represent therapeutic targets (e.g. HbA_1c_ and BMI) [12]. For example, an individual with a BMI of 35 kg/m^2^ at diagnosis might change their subtype after successful weight loss following treatment with glucagon-like peptide-1 receptor agonists (GLP1-RAs).

**Table 1:** Example of two individuals who lie between two type 2 diabetes subtypes

Variable	Individual A	Individual B
GADA	Negative	Negative
Sex	Female	Female
HbA_1c_ (mmol/mol)	65	65
HbA_1c_ (%)	8.1	8.1
BMI (kg/m^2^)	30.0	30.5
Age at onset (years)	58	58
HOMA2-B	121	121
HOMA2-IR	2.83	2.83
Subtype assignment	MARD	MOD

While recent evidence shows some differences in complications between the MOD and MARD subtypes [12], the focus is usually more on the severe SIDD and SIRD subtypes, which are further apart in terms of Euclidean distances

**Fig. 2:**
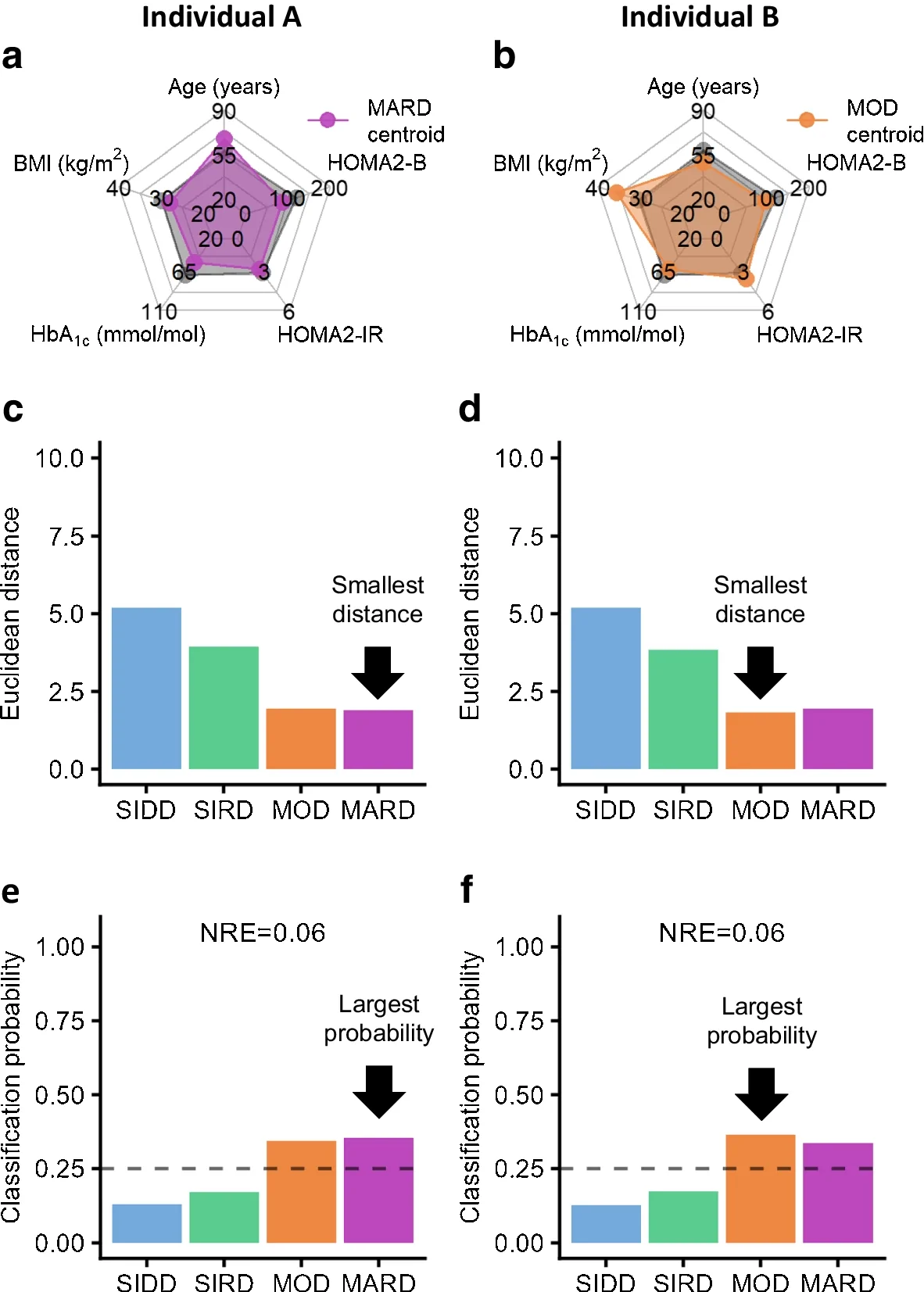
Illustrative example of different measures of classification uncertainty applied to individual A (**a**, **c**, **e**) and individual B (**b**, **d**, **f**) from Table 1. (**a**, **b**) The spider plots show that both individuals share a similar clinical profile, which does not match entirely with either MARD or MOD. (**c**, **d**) Because of their higher BMI by 0.5 kg/m^2^, individual B has a minimally smaller Euclidean distance to the MOD centroid (i.e. the typical MOD profile). (**e**, **f**) As a result, the classification probability for MOD is slightly higher than that for MARD. Because both individuals lie at the border between MOD and MARD, they have a low classification certainty, as indicated by the low classification probabilities and NRE values close to zero. This figure is available as part of a downloadable slideset

In response to this problem, the second PMDI consensus report [10] recommends that diabetes subtype assignments should always be accompanied by a measure of classification uncertainty. In principle, there are several measures that can be used to assess an individual’s classification uncertainty [35], ranging from graphical displays (e.g. spider plots) to statistical measures (e.g. Euclidean distance [8], distance ratio [22], classification probability [36, 37] and normalised relative entropy [NRE] [35]) (see Text box, ‘Measures of classification uncertainty’). Figure 2 shows an illustrative example of different measures of classification uncertainty applied to individuals A and B from Table 1.

**Figure:**
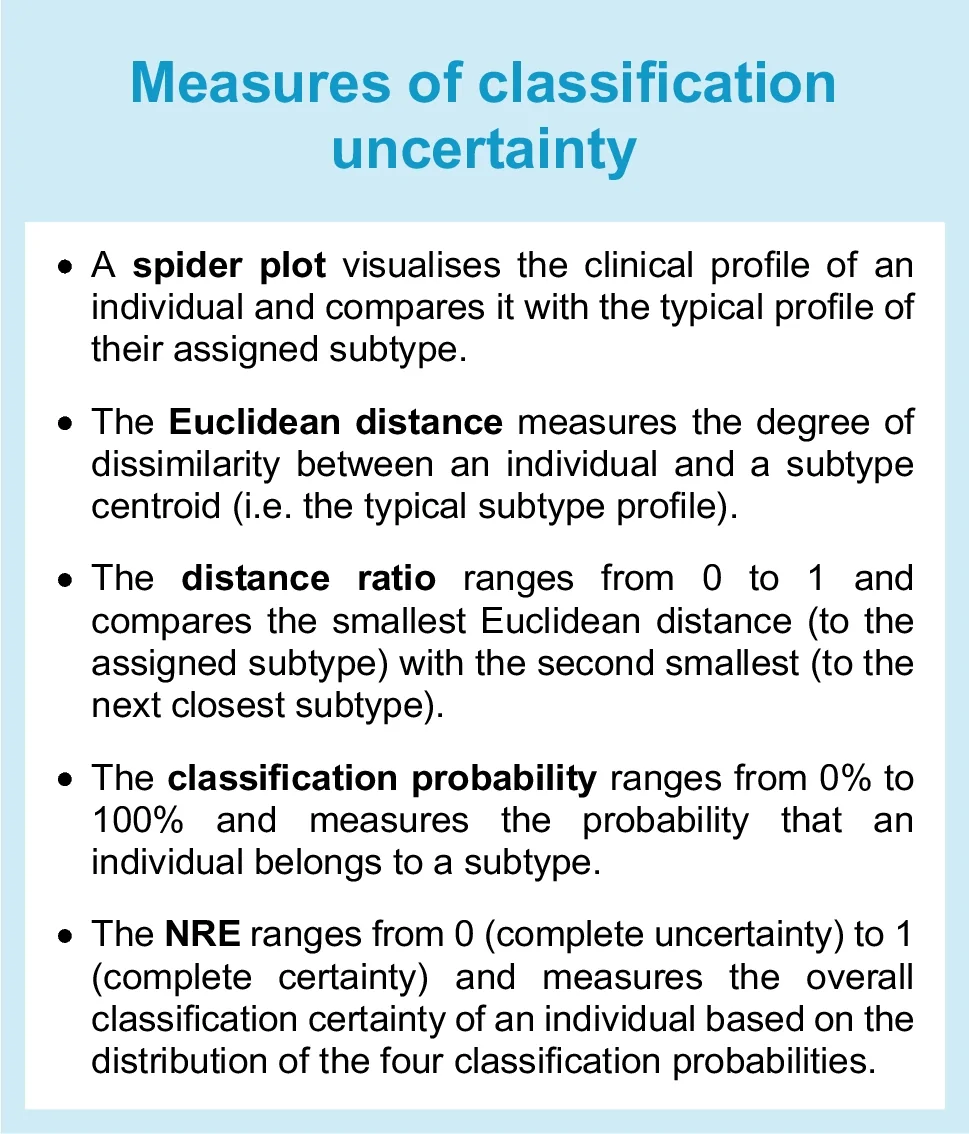

In practice, however, measures of classification uncertainty have rarely been reported in studies of the type 2 diabetes subtypes [22, 35–37]. A recent study proposed using the NRE statistic to quantify an individual’s classification uncertainty on a scale from 0 (complete uncertainty) to 1 (complete certainty) [35]. Application of this method to a cohort of 859 individuals from the German Diabetes Study revealed a low median NRE of 0.13 (95% CI 0.12, 0.14), indicating substantial classification uncertainty. While the degree of uncertainty might vary depending on the cohort and the chosen measure, current evidence shows that a certain level of classification uncertainty is inevitable [22, 35–37]. It is therefore essential to acknowledge this limitation of discrete subtyping and to routinely report uncertainty measures.

A general problem of hard subtype assignment is that, by definition, it reduces the information available in a system. Even when the classification probability for one subtype is clearly higher than the probabilities for the other subtypes, the relative magnitude of the classification probabilities still carries information about an individual’s clinical profile. Indeed, it has been suggested that the classification probabilities or Euclidean distances should be directly related to clinical outcomes, rather than the hard subtype assignment [26]. Alternatively, measures of classification uncertainty can be used to mitigate some of the information loss that occurs when comparing clinical outcomes between hard subtype assignments [35]. For example, individuals can be weighted by their NRE, such that those who are less representative of their subtype contribute less to the analysis. Another option is to use alternative prediction-based approaches for precision medicine instead of diabetes subtypes, as discussed in the next section.

### Limited prognostic and therapeutic value of subtypes

The third challenge of the subtyping approach is that the information loss due to hard subtype assignments is disadvantageous for predicting clinical outcomes (i.e. precision prognostics) [26, 34]. Instead, it would be more informative to use statistical models that directly incorporate continuous clinical features as predictors. This issue has been investigated in several studies comparing the performance of type 2 diabetes subtypes with conventional prediction models [19, 29, 38].

Aoki et al [29] compared the predictive performance of the subtypes to that of established clinical risk scores using data from the SAVOR-TIMI 53 trial, which included individuals with established CVD and long-standing diabetes (mean of 8.7 years). They employed the widely used Systematic Coronary Risk Evaluation (SCORE) [39] and the Kidney Disease: Improving Global Outcomes (KDIGO) classification [40] to predict CVD and kidney outcomes, respectively. Even though the CVD SCORE was treated as a broad ordinal variable (10 year CVD risk of ≤5%, 5–10%, 10–15%), its prognostic performance was similar to that of the subtypes (concordance index [c-index] 0.57 and 0.56 [95% CI not reported], respectively). For kidney events, prediction using the KDIGO classification clearly outperformed the subtype approach (c-index 0.62 and 0.54 [95% CI not reported], respectively). Similar results were found by Bjarkø et al [38], who assessed the predictive performance of the subtypes in a Norwegian population-based study. Using data from the Trøndelag Health Study (HUNT Study), they found that individual established risk factors (e.g. HbA_1c_, eGFR) were at least as good as the subtypes for predicting vascular complications (e.g. retinopathy, chronic kidney disease). In a re-analysis of the ADOPT [41] trial (median follow-up time of 4 years), Dennis et al [19] found that age at diagnosis alone was as informative as the subtype classification for predicting glycaemic disease progression (*R*^*2*^=0.09 and 0.08, respectively).

Beyond precision prognostics, Dennis et al [19] critically examined the subtypes’ potential for precision treatment. They found that the subtypes differed modestly in their response to glucose-lowering drugs in the ADOPT trial, which compared metformin, sulfonylurea and thiazolidinedione treatment. However, a model that directly used the clustering variables as predictors, even without HOMA2 measures, explained individual treatment response substantially better. When predicting response to sulfonylureas, the model explained 33% of the variability in treatment response, compared with 20% for the subtype-based approach. Similar results were found when predicting response to thiazolidinedione treatment (*R*^*2*^_model_=0.32 vs *R*^*2*^_subtypes_=0.17) and metformin treatment (*R*^*2*^_model_=0.35 vs *R*^*2*^_subtypes_=0.15). To further compare these two approaches, Dennis et al [19] performed an external validation using the RECORD trial [42] (mean follow-up time of 5.5 years), which investigated the same medications. They found that treatment recommendations based on the prediction model yielded greater benefits than those based on subtypes, with an additional HbA_1c_ reduction of 3.9 mmol/mol (0.36 percentage points) compared with 1.8 mmol/mol (0.16 percentage points).

A limitation of these findings is that they are based on selective populations from randomised clinical trials, which might not be representative of the general type 2 diabetes population. The ADOPT trial [41] included recently diagnosed individuals without prior pharmacological therapy and with fasting plasma glucose levels of 7.0–10.0 mmol/l. The RECORD trial [43] included individuals on metformin or sulfonylurea monotherapy and with HbA_1c_ levels of 53–75 mmol/mol (7–9%). As a result, the average HbA_1c_ value in the identified SIDD subtype was substantially lower (median: ADOPT, 67 mmol/mol [8.3%]; RECORD, 72 mmol/mol [8.7%]) [19] than in the original study by Ahlqvist et al [8] (mean: ANDIS, 102 mmol/mol [11.5%]). Moreover, differences in the risk of kidney disease, repeatedly shown in other cohorts [8, 14, 16], could not be replicated clearly in these cohorts [19]. A possible explanation is that these cohorts excluded individuals with baseline renal impairment, resulting in a relatively high median eGFR at baseline even for the SIRD subtype (ADOPT, 90 ml/min per 1.73 m^2^; RECORD, 97 ml/min per 1.73 m^2^) [19]. Finally, both ADOPT [41] and RECORD [43] excluded individuals with comorbidities such as heart failure and did not investigate more-recent therapies such as sodium–glucose cotransporter 2 inhibitors (SGLT2i) and GLP1-RAs.

An overarching limitation of the above studies is that they involved retrospective analyses of cohorts that were not originally designed to investigate the type 2 diabetes subtypes [44]. To our knowledge, there has been only one prospective clinical trial that was explicitly designed to assess differences in treatment response among the diabetes subtypes [45]. In this trial, Dwibedi et al [45] assessed whether individuals with SIDD (*n*=126) and SIRD (*n*=113) respond differently to glucose-lowering treatment with GLP1-RA and SGLT2i. The study was designed to detect a treatment effect difference (i.e. a treatment-by-subgroup interaction) of 3.5 mmol/mol (0.32 percentage points) in 6 month HbA_1c_ with a statistical power of 80%. However, their results showed no differential treatment response (i.e. no statistically significant interaction), with GLP1-RA being more effective than SGLT2i in both SIDD and SIRD (HbA_1c_ benefit of 8.6 mmol/mol [0.79 percentage points] and 7.8 mmol/mol [0.71 percentage points], respectively). Instead, they found a statistically significant interaction between treatment and baseline HbA_1c_, suggesting that GLP1-RAs are increasingly more effective than SGLT2i at higher baseline HbA_1c_ levels. In other words, their results again suggested that continuous pathophysiological variables might be more useful than diabetes subtypes for predicting glucose-lowering treatment response. However, there are two major limitations to this clinical trial. First, the strict eligibility criteria (e.g. requirement for metformin monotherapy) excluded individuals with more progressive diabetes and resulted in SIDD and SIRD groups that were less distinct from each other. For example, the mean baseline HbA_1c_ level of individuals with SIDD and SIRD was 57.5 mmol/mol (7.4%) and 53.4 mmol/mol (7.0%), respectively. Second, the trial only had a follow-up time of 6 months and focused on glycaemic outcomes. However, proponents of subtyping typically focus on the importance of SGLT2i and GLP1-RA treatment for reducing cardio-renal complications, rather than glucose, in individuals with SIRD [12, 46]. More detailed considerations of potential treatment options for the diabetes subtypes based on their pathophysiology have been provided in other reviews [9, 46].

From a methodological perspective, the aforementioned findings regarding the potentially limited prognostic and therapeutic value of subtypes are not surprising, given that clustering methods (analogous to unsupervised learning) are not designed to achieve the best possible prediction of future clinical outcomes [34]. Instead, they focus on cross-sectional data to identify homogenous groups of individuals with distinct clinical features (i.e. precision diagnosis). If interest lies in precision prognostics or precision treatment, then dedicated prediction methods (analogous to supervised learning) might be preferable, as those are designed to achieve an optimal prediction of future clinical outcomes.

## Implications for type 2 diabetes precision medicine

### Individualised prediction models as an alternative to subtyping

Building on the work above [19], Dennis and colleagues continued to develop and apply the ‘individualised prediction’ framework for precision treatment of type 2 diabetes [47–51]. In this framework [47], the aim is to use simple clinical features to directly model treatment response (i.e. precision treatment) rather than to identify distinct aetiologies of diabetes (i.e. precision diagnosis). The goal is to identify the optimal treatment for an individual based on their clinical features at a particular point in time, without requiring an explicit subtype assignment. To ensure applicability in clinical practice, Dennis [47] emphasised the benefit of using routinely available clinical features, such as age, BMI and eGFR, to provide a low-cost equitable precision medicine approach. So far, applications of the individualised prediction approach have focused on treatment selection models (or ‘treatment algorithms’) for second- and third-line glucose-lowering drugs [48, 49, 51].

**Fig. 3:**
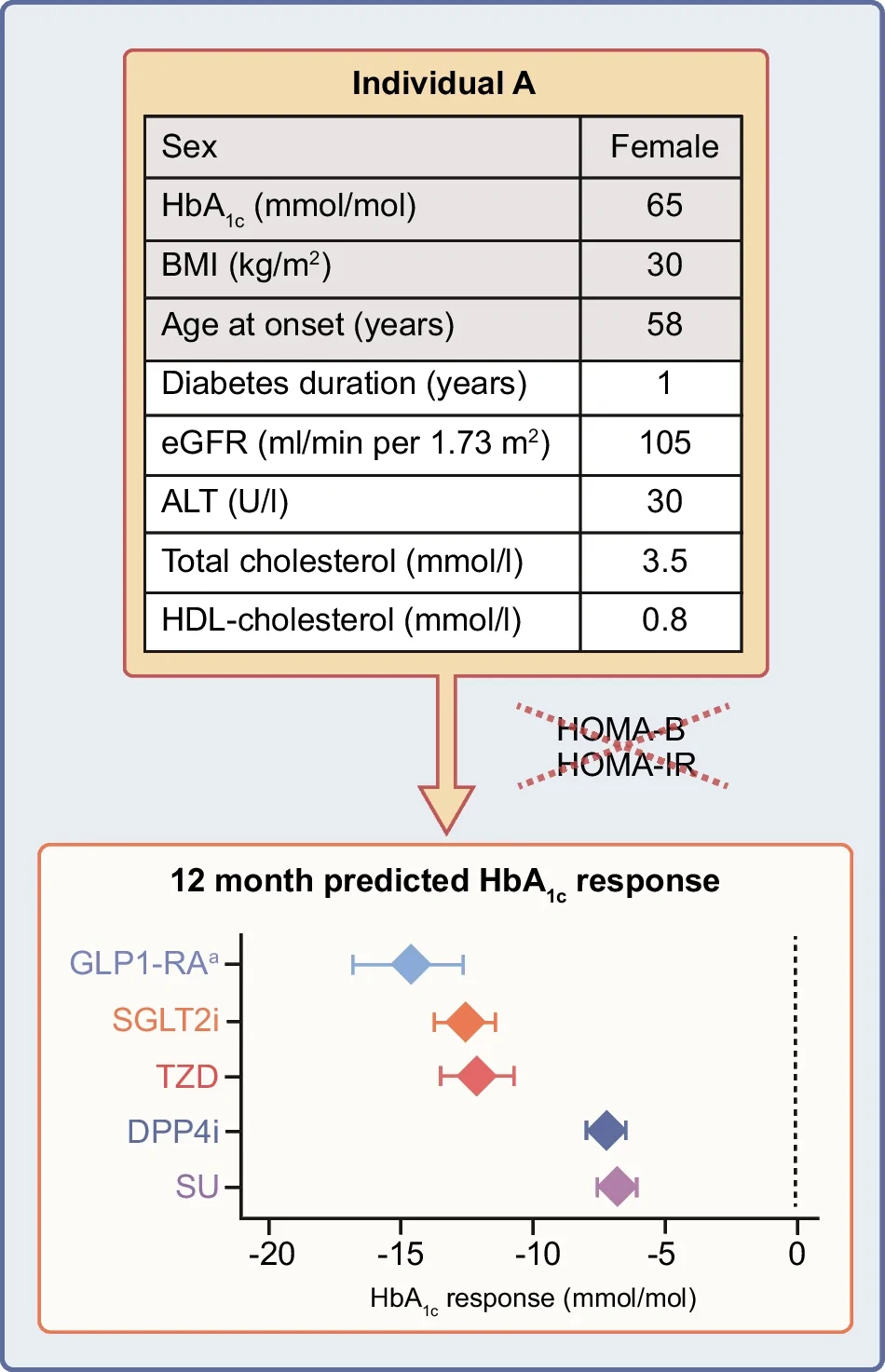
Illustrative example of the five-drug-class model [51] applied to individual A from Table 1. Similar to the subtyping approach, the model requires as input an individual’s sex, HbA_1c_, BMI and age at treatment decision (grey features in table). While the model does not require HOMA2-B and HOMA2-IR, it depends on five additional routine clinical features (white features in table): diabetes duration, eGFR, ALT, total cholesterol and HDL-cholesterol. For individual A, the model predicts that treatment with GLP1-RA, SGLT2i or thiazolidinedione (TZD) will achieve the highest glucose-lowering response. While a slightly larger benefit is predicted for GLP1-RA, the overlapping CIs suggest that SGLT2i and TZD may be similarly effective. In such a setting of clinical equipoise, a shared decision-making process that incorporates patient preferences (e.g. oral medication vs injectable) could support treatment selection [5]. Note that, because the clinical variables are modelled continuously, small-value changes (e.g. 0.5 kg/m^2^ higher BMI of individual B) have little effect and lead to the same therapies being predicted as optimal (data not shown). ^a^Model development excluded semaglutide. DPP4i, dipeptidyl peptidase 4 inhibitor; SU, sulfonylurea. This figure is available as part of a downloadable slideset

A recent major advance within this framework is the development of a ‘five-drug-class model’ (available at www.diabetesgenes.org/t2-treatment/) [51]. This model allows simultaneous prediction of an individual’s 12 month HbA_1c_ response to any of the five glucose-lowering drug classes commonly prescribed after metformin in primary care: SGLT2i, GLP1-RAs, dipeptidyl peptidase-4 inhibitors, sulfonylureas and thiazolidinediones. The model was developed using observational primary care data from the UK Clinical Practice Research Datalink (CPRD), comprising more than 100,000 drug initiations. Nine routine clinical features (age, duration of diabetes, sex, HbA_1c_, BMI, eGFR, HDL-cholesterol, total cholesterol, alanine aminotransferase [ALT]) were included as predictors of differential treatment response. An illustrative example of the five-drug-class model applied to individual A from Table 1 is shown in Fig. 3.

Model validation was performed in an independent CPRD validation cohort as well as in three clinical trials, including a three-way crossover trial [52]. In the CPRD cohort, applying a clinically relevant threshold of ≥3 mmol/mol (0.27 percentage points), the model predicted optimal therapies for the majority of initiations, with 54.9% (*n*=116,525) having one or two drug classes predicted to provide a greater HbA_1c_ reduction than other drug classes. The most frequently predicted optimal therapies for HbA_1c_ reduction were GLP1-RAs (optimal in 33.4% of treatment initiations), SGLT2i (28.9%) or sulfonylureas (27.6%) [51]. Treatment in line with these recommendations was associated with a mean additional HbA_1c_ reduction of 5.0–5.3 mmol/mol (0.46–0.48 percentage points) compared with non-optimal treatment. The model-predicted HbA_1c_ benefits showed good calibration in external validation. Absolute observed HbA_1c_ benefits were smaller in the more selective clinical trial populations (mean additional HbA_1c_ reductions of 1.9–2.4 mmol/mol [0.17–0.22 percentage points]) where mean baseline HbA_1c_ was lower than in the real-world datasets. CPRD analysis suggested following the model recommendations was also associated with a 38% lower risk of glycaemic failure (reaching an HbA_1c_ ≥69 mmol/mol [8.5%]) up to 5 years post therapy initiation, although long-term benefits remain to be confirmed in external data. Overall, these initial findings position the individualised prediction approach as a promising alternative to subtype-based stratification for precision treatment selection.

Nevertheless, there are also limitations to this individualised prediction approach. First, the focus lies on optimising treatment selection for lowering glucose rather than preventing cardio-renal complications [53]. However, validation of the five-drug-class model in observational CPRD data also showed an associated lower risk of cardiovascular and renal complications after 5 years, likely reflective of the frequent recommendation of GLP1-RAs and SGLT2i [51]. Second, the frequent recommendation of these novel medications (62.4% of treatment initiations in CPRD were recommended GLP1-RA or SGLT2i) could lead to increased healthcare costs. However, treatment recommendations were often not limited to a single drug class (76.4% of initiations in CPRD had two or more optimal drug classes) and the model allows for flexible deployment by omitting treatments that might not be available (e.g. due to costs or contraindications) [51]. Third, prediction models developed from observational data come with their own methodological challenges such as overfitting, confounding and incomplete data [54, 55]. While initial results seem reassuring [51, 56], careful validation [57] and calibration [58] of these models remains crucial to support decision-making in clinical practice.

### The role of subtypes in type 2 diabetes precision medicine

One main advantage of diabetes subtypes over individualised prediction models is that they may provide insights into the underlying pathophysiology of the disease [31]. Ahlqvist et al [59] emphasised that, even though they employed data-driven clustering methods, their subtyping approach was not hypothesis free. Their variable selection reflected major pathways in the development of type 2 diabetes (e.g. insulin resistance) and captured disease heterogeneity in a clinically meaningful way. In principle, clustering methods can help identify subgroups with distinct clinical features that would otherwise be clouded out by the majority. This can then prompt investigation into which pathophysiological mechanisms may have caused this subgroup to emerge. For example, a recent study found that individuals with SIRD had a higher intrahepatic lipid content than the other subtypes; this, in turn, was associated with a higher cardiovascular risk [60]. The notion that the subtypes differ in terms of their aetiology is also supported by genetic evidence [9, 25, 61]. For example, unlike the other subtypes, SIRD is not associated with polygenic risk scores for impaired insulin secretion [62]. Given such pathophysiological insights, Ahlqvist et al [31] have argued that the subtypes provide ‘a more holistic view of type 2 diabetes’ than individualised prediction models.

In terms of its underlying assumptions, the subtype framework differs from the ‘palette model’ of type 2 diabetes heterogeneity [3]. The latter emphasises that disease heterogeneity arises from multiple component pathways that jointly contribute to an individual’s phenotype, often resulting in a ‘mixed aetiology’. While Ahlqvist et al [25] acknowledge that diabetes is caused by many overlapping mechanisms, they make the assumption that most individuals are predominantly affected by one pathway. A key advantage of the subtyping approach is that discrete subtypes are easy to communicate and can provide a broad range of clinical information [9, 47]. Rather than having to use a different prediction model for each clinical outcome, the subtype assignment can feasibly provide an overview based on established subtype-specific risks of complications. That said, including a measure of classification uncertainty is advisable to gauge how applicable subtype-specific predictions are for a given individual [35].

When discussing the feasibility of the subtyping approach, it is important to also consider logistical obstacles to implementation in clinical practice. While a user-friendly web application has recently been developed [63], subtype assignment requires HOMA2 indices of insulin resistance and insulin secretion [8], particularly for correctly identifying individuals with SIRD [25]. Calculating these HOMA2 values introduces additional computational complexity, as it requires a separate (although freely available) calculator program [11]. The more fundamental challenge is that the calculation requires fasting insulin or C-peptide measurements as input. However, these are not routinely collected in clinical practice, making a widespread implementation challenging at present [7, 21, 22, 24]. In addition, access to these measurements may be more restricted in low-income and middle-income countries with resource-constrained healthcare systems [64]. However, as C-peptide measurements are relatively inexpensive (e.g. compared with novel GLP1-RA medications), a broader rollout could be possible in the future [12].

## Conclusions and future directions

Type 2 diabetes subtyping has made important contributions towards precision medicine but it also comes with several methodological limitations. It is important to be mindful of challenges such as low classification certainty and practical implementation barriers due to use of non-routine biomarkers. Individualised prediction models offer an actionable alternative by providing personalised treatment recommendations based on routine clinical features. However, they do not provide insights into the underlying pathophysiology of the disease and thus might not paint a complete picture of diabetes heterogeneity. As such, these two approaches can be considered as complementary, each serving different purposes in precision medicine [59, 61, 65].

From a methodological perspective [66], clustering methods (analogous to unsupervised learning) aim to delineate groups with distinct clinical features by minimising within-cluster differences. In contrast, prediction methods (analogous to supervised learning) are designed to achieve the best possible prediction of future outcomes by minimising prediction errors. In other words, these two approaches optimise for different parameters, which naturally leads to different use cases [34]: for precision treatment, individualised prediction models might be preferable, as advocated by the second PMDI consensus report [10]; for precision diagnostics, diabetes subtyping can be employed to gain insights into patterns of phenotypic variability and pathophysiology; and for precision prognostics, diabetes subtyping can be supplemented by outcome-specific prediction models, such as SCORE2-Diabetes for cardiovascular outcomes [67].

For precision prevention, a distinct set of subtypes among people at risk of type 2 diabetes has been proposed [68–70]. The methodological limitations of type 2 diabetes subtypes outlined above directly transfer to these subtypes of people at risk of type 2 diabetes and, more broadly, to any data-driven clustering approach. By raising awareness of these challenges through this narrative review, we hope to enable a more careful application of diabetes subtyping in research and clinical practice.

Regarding the challenge of classification uncertainty, the argument can be extended to any type of diabetes classification that uses a threshold. For example, an individual with an HbA_1c_ of 48 mmol/mol (6.5%) would be classified as having diabetes, whereas an identical individual with an HbA_1c_ of 46 mmol/mol (6.4%) would not [1]. Similarly, a very small difference in GADA titre could determine whether a person is ‘autoantibody positive’ and thus considered to have type 1 or type 2 diabetes [71]. For a detailed discussion of the challenges associated with delineating type 1 and type 2 diabetes, see Gale [71], which provides a useful parallel to our discussion of type 2 diabetes subtypes.

As for the individualised prediction approach, future research could consider models with multivariate outcomes to allow for simultaneous prediction of several outcomes (e.g. HbA_1c_, kidney function, cardiovascular risk). In the future, incorporation of prediction models into decision support systems coupled to electronic medical records could provide a feasible implementation for clinical practice. At the same time, the predicted optimal treatment may not necessarily be consistent across different outcomes, making it potentially difficult to derive treatment recommendations for an individual in such a scenario.

Reflecting on precision medicine more broadly, the second PMDI consensus report [10] raises concerns about a lack of adequate validation studies. In particular, there is a lack of prospectively designed clinical trials to validate proposed precision medicine approaches. One exception is the clinical trial mentioned above, which was explicitly designed to assess differential treatment responses of individuals classified as SIDD and SIRD [45]. Another notable example is the TriMaster study [52], a three-way crossover trial that demonstrated differential responses to three glucose-lowering medications depending on individuals’ kidney function and BMI. However, such precision medicine trials remain scarce, and the PMDI consensus report concludes that the clinical translation of precision medicine in diabetes care is, at present, ‘largely aspirational’ [10].

Consequently, an important objective for future research is to generate high-quality evidence from appropriately designed clinical trials [72–74]. For example, repeated crossover trials (or ‘*N*-of-1-trials’), in which each individual receives at least one treatment twice, are particularly useful to address precision medicine questions [75, 76]. This trial design is uniquely suited to demonstrate treatment effect heterogeneity. In other words, it enables researchers to determine whether, and to what extent, individuals truly differ in their responses to different glucose-lowering medications [72, 77]. While no such trials have yet been conducted for glucose-lowering medications, a successful example has recently been published for blood-pressure-lowering treatment [78].

Beyond establishing treatment heterogeneity, the second prerequisite for precision medicine is the identification of clinical features that alter treatment response [72]. For example, the five-drug-class model suggests that routinely collected features such as BMI, eGFR and cholesterol can identify people who would benefit more from one treatment than another [51]. To evaluate such precision medicine approaches, a pragmatic strategy would be a randomised trial in which one group of healthcare providers uses the treatment algorithm and the other group does not [47]. Such a design would enable a comparison of the precision medicine approach with current standards of care in a routine clinical practice setting [47, 79]. Regardless of the study design, future research should adhere to the BePRECISE reporting guidelines [80] to facilitate evaluation of the clinical utility of proposed precision medicine strategies.

In conclusion, this narrative review highlights three key methodological challenges of type 2 diabetes subtypes: (1) uncertainty about the existence of four discrete subtypes; (2) low classification in subtype assignment; and (3) limited evidence for prognostic and therapeutic value compared with other approaches. As an alternative, we discuss individualised prediction models that avoid some of the limitations of discrete subtyping and provide personalised treatment recommendations based on routinely collected clinical features.

## Supplementary Information

Below is the link to the electronic supplementary material.

Slideset of figures (PPTX 358 KB)

## Abbreviations

ALT: Alanine aminotransferase
ANDIS: All New Diabetics in Scania
c-index: Concordance index
CPRD: Clinical Practice Research Datalink
GLP1-RA: Glucagon-like peptide-1 receptor agonist
KDIGO: Kidney Disease: Improving Global Outcomes
MARD: Moderate age-related diabetes
MOD: Moderate obesity-related diabetes
NRE: Normalised relative entropy
PMDI: Precision Medicine in Diabetes Initiative
SAID: Severe autoimmune diabetes
SCORE: Systematic Coronary Risk Evaluation
SGLT2i: Sodium–glucose cotransporter 2 inhibitor
SIDD: Severe insulin-deficient diabetes
SIRD: Severe insulin-resistant diabetes
